# *Tetraselmis suecica* F&M‐M33 growth is influenced by its associated bacteria

**DOI:** 10.1111/1751-7915.12865

**Published:** 2017-11-03

**Authors:** Natascia Biondi, Giulia Cheloni, Liliana Rodolfi, Carlo Viti, Luciana Giovannetti, Mario R. Tredici

**Affiliations:** ^1^ Department of Agrifood Production and Environmental Sciences (DISPAA) University of Florence Piazzale delle Cascine 24 50144 Florence Italy; ^2^ Genexpress Laboratory University of Florence Via della Lastruccia 14 50019 Sesto Fiorentino Florence Italy

## Abstract

Algal cultures are usually co‐cultures of algae and bacteria, especially when considering outdoor mass cultivation. The influence of associated bacteria on algal culture performance has been poorly investigated, although bacteria may strongly affect biomass (or derived product) yield and quality. In this work, the influence on growth and productivity of *Tetraselmis suecica* F&M‐M33 of bacterial communities and single bacterial isolates from the algal phycosphere was investigated. Xenic laboratory and outdoor cultures were compared with an axenic culture in batch. The presence of the bacterial community significantly promoted culture growth. Single bacterial isolates previously found to be strictly associated with *T. suecica* F&M‐M33 also increased growth compared with the axenic culture, whereas loosely associated and common seawater bacteria induced variable growth responses, from positive to detrimental. The increased growth was mainly evidenced as increased algal biomass production and cell size, and occurred after exhaustion of nutrients. This finding is of interest for biofuel production from microalgae, often attained through nutrient starvation processes leading to oil or carbohydrate accumulation. As axenic *T. suecica* F&M‐M33 showed a similar growth with or without vitamins, the most probable mechanism behind bacterial positive influence on algal growth seems nutrient recycling.

## Introduction

Microalgal biotechnology, in recent years, has sparked great interest, initially focused on the potential use of microalgal biomass as feedstock for biofuel production and then also as a source of alternative foods and food ingredients (Vanthoor‐Koopmans *et al*., 2013). For algae to become real players in the market of biofuels and food commodities, production of huge amounts of biomass at low cost is necessary. To achieve this goal, besides lowering the operational and capital costs of algal biomass production at large scale (Tredici *et al*., 2015, 2016), improvement of culture productivity is required. In this respect, one of the aspects of algal cultivation that has been largely disregarded is the role played by the co‐cultivated bacteria (Kim *et al*., 2014; Cho *et al*., 2015). Only in few niche applications, algal cultures are axenic, while in all mass cultivation systems (including laboratory vessels and photobioreactors), algae form a consortium with bacteria which may enter as airborne contaminants (Chini Zittelli *et al*., 2013), or brought with the water and the sea salts used to prepare artificial seawater, or finally are associated with the algal cells since the isolation process that led from the original environmental sample to the monoalgal culture (Biondi *et al*., 2017).

In analogy to plant rhizosphere, the area extending out of the algal cell in which interactions with bacteria take place has been termed ‘phycosphere’ (Bell and Mitchell, 1972). Algae–bacteria interactions in the phycosphere can vary depending on the main growth‐limiting factor and the same bacteria can either stimulate or inhibit algal growth, depending on the physiological state of the alga (Ramanan *et al*., 2016). For example, it has been observed that often under phosphorus limitation bacteria compete with algae for this nutrient‐depressing algal growth, while under nitrogen limitation the effect of bacteria on algal growth may be neutral or positive, due to a balance between nitrogen release through organic matter degradation and nitrogen immobilization, while in a medium with no nutrient limitation bacteria may stimulate algal growth by providing CO_2_ (Brussard and Riegmann, 1998; Danger *et al*., 2007; Amin *et al*., 2012; Ramanan *et al*., 2016). This different behaviour may play an important role during the starvation of algal cultures, a condition often applied to favour the accumulation of storage products (oil/carbohydrate) for biofuel production (Rodolfi *et al*., 2009; Bondioli *et al*., 2012; Yao *et al*., 2012; Garnier *et al*., 2016). Bacteria, besides inhibiting algal growth due to competition for nutrients, may also release harmful compounds, such as algicidal molecules or exoenzymes (Amin *et al*., 2012; Natrah *et al*., 2014; Cooper and Smith, 2015; Fuentes *et al*., 2016; Ramanan *et al*., 2016).

Stimulation of algal growth by bacterial communities or bacterial isolates has also been widely reported (Suminto and Hirayama, 1997; Arora *et al*., 2012; Amin *et al*., 2015; Cho *et al*., 2015; Fuentes *et al*., 2016). This may occur through different mechanisms, among which nutrient regeneration, release of trace elements and vitamins (particularly vitamin B_12_) or of stimulatory compounds, enrichment of CO_2_ and consumption of excess O_2_, or scavenging of reactive oxygen species in the algal microenvironment (Mouget *et al*., 1995; Croft *et al*., 2005; Hünken *et al*., 2008; Natrah *et al*., 2014; Cooper and Smith, 2015; Fuentes *et al*., 2016; Park *et al*., 2017). Addition of selected bacterial strains in mass cultures has been shown to have a stabilizing effect, improving microalgal production reliability (Fukami *et al*., 1997).

Usually, algae favour chemoheterotrophic bacteria growth thanks to the release of extracellular compounds, which may vary according to the associated bacterium that may in turn release factors influencing the alga (Bruckner *et al*., 2008; Natrah *et al*., 2014). Many bacteria show a chemotactic response, often regulated by amino acids, towards algal culture filtrates or exudates (Bell and Mitchell, 1972; Barbara and Mitchell, 2003; Stocker and Seymour, 2012). Besides algae releasing stimulatory substances (Terekhova *et al*., 2009; Natrah *et al*., 2014), there are many able to produce bactericidal or bacteriostatic compounds (Tredici *et al*., 2009). These latter are of huge biotechnological interest in the search for new antibiotics (Senhorinho *et al*., 2015; Falaise *et al*., 2016).

Few studies (Nicolas *et al*., 2004; Arora *et al*., 2012; Biondi *et al*., 2017) have investigated the bacterial communities associated with cultures of the marine microalga *Tetraselmis* (Chlorodendrophyceae, Chlorophyta) and the influence of these communities on *Tetraselmis* growth (Meseck *et al*., 2007; Arora *et al*., 2012; Park *et al*., 2017). This microalga is one of the few actually in the market, as it is widely used in aquaculture (Abiusi *et al*., 2014; Tredici *et al*., 2009; Tulli *et al*., 2012; Muller‐Feuga, 2013). *Tetraselmis* also represents a possible feedstock for biofuel production (Rodolfi *et al*., 2009; Bondioli *et al*., 2012; Biondi *et al*., 2016), cosmetic applications (Pertile *et al*., 2010) and also food as the species *Tetraselmis chuii* has recently been approved as novel food in the EU (AECOSAN, 2014).

The purpose of this work was to investigate the effect of the associated bacteria on the growth of the marine microalga *Tetraselmis suecica* F&M‐M33. Bacterial communities from a laboratory and an outdoor mass culture and single bacteria isolated from these communities and reassociated with the axenic alga were tested under high and low nutrient concentrations and in media with or without of vitamins.

## Results

Two bacterial communities were investigated for their effect on *T. suecica* F&M‐M33 growth: one associated with a laboratory xenic algal culture (LAB) always maintained and cultivated under axenic conditions and the other associated with an outdoor (and thus always exposed to contaminants) algal culture (OUT) carried out in semicontinuous in a photobioreactor and sampled in autumn after about 8 months of continuous operation. For the composition of the communities, see Biondi *et al*. (2017). An axenic culture (AX) was set up for comparison. The cultures were performed in batch using 500 ml bubbled tubes kept under continuous illumination.

The effect on algal growth of the addition to the axenic culture of single bacteria isolated from *T. suecica* F&M‐M33 phycosphere (LAB and OUT cultures) and of two environmental isolates (from seawater) was also evaluated. The bacterial strains were individually co‐cultivated with the axenic alga in batch in 50 ml bubbled tubes under continuous illumination and the growth of co‐cultures was compared with that of AX and LAB cultures.

The effect of vitamins on growth of *T. suecica* F&M‐M33 in the absence (AX) and presence of the whole bacterial community (LAB) or of single bacteria was finally evaluated. Tests were carried out in 60 ml bottles incubated in an orbital shaker under light/dark cycles.

### Effect of total bacterial community on the growth of *Tetraselmis suecica* F&M‐M33

**Figure 1:**
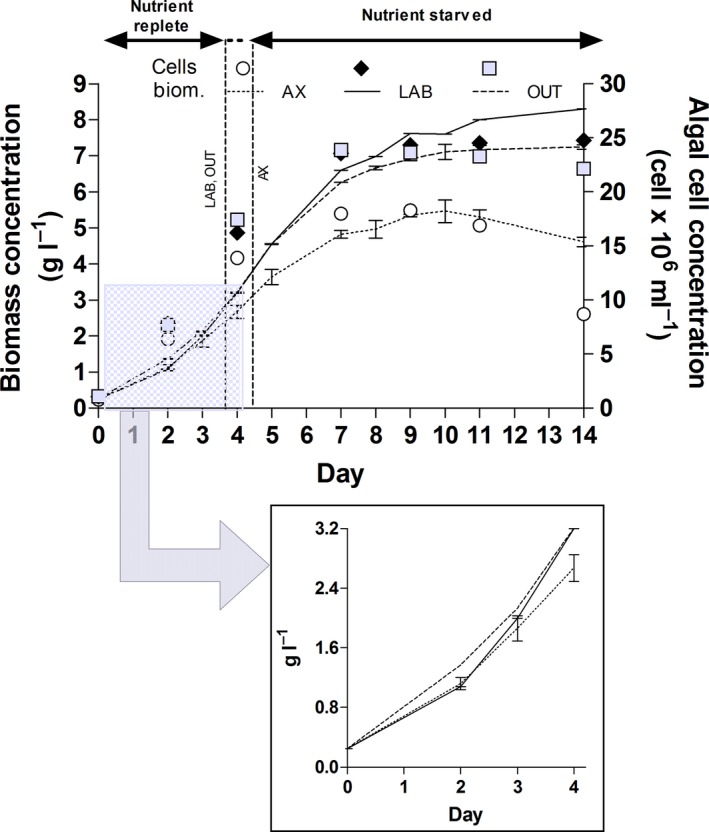
Growth curves of axenic and xenic *Tetraselmis suecica* F&M‐M33 cultures in 500 ml bubble tubes expressed as biomass dry weight and algal cell concentration. The inset evidences growth in the first four days until exhaustion of nutrients in the medium. The day at which the cultures reached a biomass growth of 2.5 g l^−1^ is indicated by the vertical dashed lines. AX, axenic culture; LAB, xenic culture with laboratory bacterial community; OUT, xenic culture with bacterial community sampled in autumn from an eight month outdoor culture.

Growth of an axenic culture (AX) and of cultures associated with a laboratory (LAB) or an outdoor (OUT) bacterial community was compared in 500 ml tubes. Biomass concentration in the axenic culture reached its maximum (5.5 g l^−1^) after 10 days of growth and then started to decrease (Fig. 1). The same trend was observed for the algal cell number (maximum value 18.6 × 10^6^ cell ml^−1^). Growth was significantly (*P* < 0.05) higher both in LAB and in OUT. While in LAB both dry weight and algal cell number were still increasing, although very slowly, at the end of the experiment (reaching 8.3 g l^−1^ and 24.8 × 10^6^ cell ml^−1^), in OUT biomass concentration reached a plateau at day 11 (7.2 g l^−1^), when algal cell number was already decreasing (maximum of 23.9 × 10^6^ cell ml^−1^ at day 7). Bacterial concentration (determined by both plate and direct count) increased of 24–25 times in LAB and of 5 times in OUT at the end of the experiment: from 7.8 to 196 × 10^6^ CFU ml^−1^ (plate count) and from 7.9 to 207 (direct count) × 10^6^ cell ml^−1^ in LAB and from 4.5 to 27 × 10^6^ CFU ml^−1^ (plate count) and from 5 to 29 × 10^6^ cell ml^−1^ (direct count) in OUT. No bacterial growth was observed in AX cultures.

**Figure 2:**
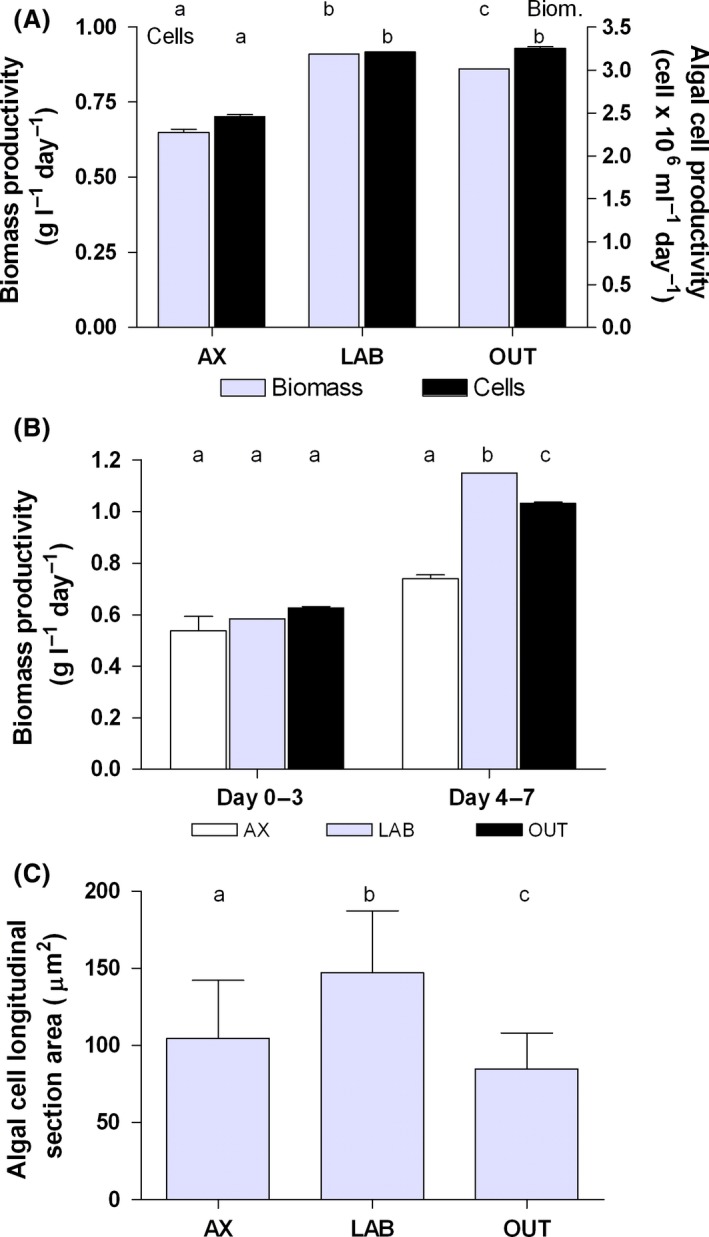
Biomass and algal cell productivities during the active growth phase (days 0–7) (A), biomass productivity split between the period of nutrient sufficiency (days 0–3) and nutrient depletion (days 4–7) (B), and algal cell dimension expressed as longitudinal section area (day 7) (C) of an axenic and two different xenic (LAB and OUT) *Tetraselmis suecica* F&M‐M33 cultures grown in 500 ml bubble tubes. Error bars indicate standard deviation. The same letter for the same group of data indicates non‐significant difference (*P* > 0.05).

The theoretical (i.e. expected according to nutrients provided at the beginning of the batch) biomass concentration (2.5 g l^−1^) was reached by LAB and OUT in 3.6 days. AX, instead, reached the theoretical biomass concentration after 4.3 days (Fig. 1 inset). Considering nitrogen as 10% of biomass, 2.5 g l^−1^ is the growth that can be obtained maintaining this N content in the biomass and thus a composition typical of cells grown under optimal conditions. In the fourth day, the biomass concentration reached by the axenic culture was lower but not significantly different (*P *> 0.05). At the end of the experimental period (14 days), after a prolonged nutrient depletion experienced by all the cultures, the difference between LAB and OUT with the axenic culture strongly increased becoming significant (*P* < 0.05) (Fig. 1). OUT reached a significantly lower (*P* < 0.05) biomass concentration and algal cell number compared with LAB (Fig. 1). After 7 days (end of the active growth phase), productivities in terms of dry biomass were similar for LAB and OUT (about 0.9 g l^−1^ day^−1^) and much lower for AX (0.7 g l^−1^ day^−1^); a similar trend was observed for productivity expressed as cell number (Fig. 2A). In the first three day period (Fig. 2B), in which nutrients were fully available for growth, biomass productivity of LAB and OUT was higher (+9% and +17%, respectively) than that of AX, but not significantly different (*P* > 0.05). Instead, from day 4 to day 7 (Fig. 2B), when nutrients had been exhausted and cultures were starving, biomass productivities of both LAB (+55%) and OUT (+40%) largely exceeded that of AX. The productivity of LAB was significantly higher than that of OUT (*P* < 0.05).

In the AX culture, *T. suecica* F&M‐M33 cells at the end of the active growth phase, besides being lower in number compared with the other two cultures (Fig. 1), had an average size significantly (*P* < 0.05) smaller than that of LAB and bigger than that of OUT (Fig. 2C). LAB also had significantly bigger cells than OUT culture. Cell size increase occurred between day 4 and day 7 (+35% for AX and OUT and +134% for LAB), when nutrients (especially nitrogen) were becoming limiting and the cultures strongly photolimited.

At the beginning of the trial, all the cultures showed a similar biochemical composition (proteins 40%–47% of dry biomass, carbohydrates 9%–12%, lipids 21%–24%, ashes 16%–18%), typical of *T. suecica* F&M‐M33 biomass grown in a nutrient‐replete medium (high protein and low carbohydrate content) (see Abiusi *et al*., 2014 for typical composition). At the end of the trial (day 14), when nitrate nitrogen was completely exhausted in all the cultures, all the biomasses showed a composition typical of nutrient‐starved conditions (high carbohydrate and low protein content) (see Bondioli *et al*., 2012 for starved biomass composition). The composition was similar in LAB and OUT (proteins 17%–18% of dry biomass, carbohydrates 37%–43%, lipids 16%–18%, ashes 17%–18%), while AX was richer in proteins and lipids (proteins 24%, carbohydrates 32%, lipids 22%, ashes 16%). At the end of the active growth period (day 7), the composition was intermediate with an almost equivalent protein and carbohydrate content in all the cultures.

### Effect of single bacteria on *Tetraselmis suecica* F&M‐M33 growth

**Figure 3:**
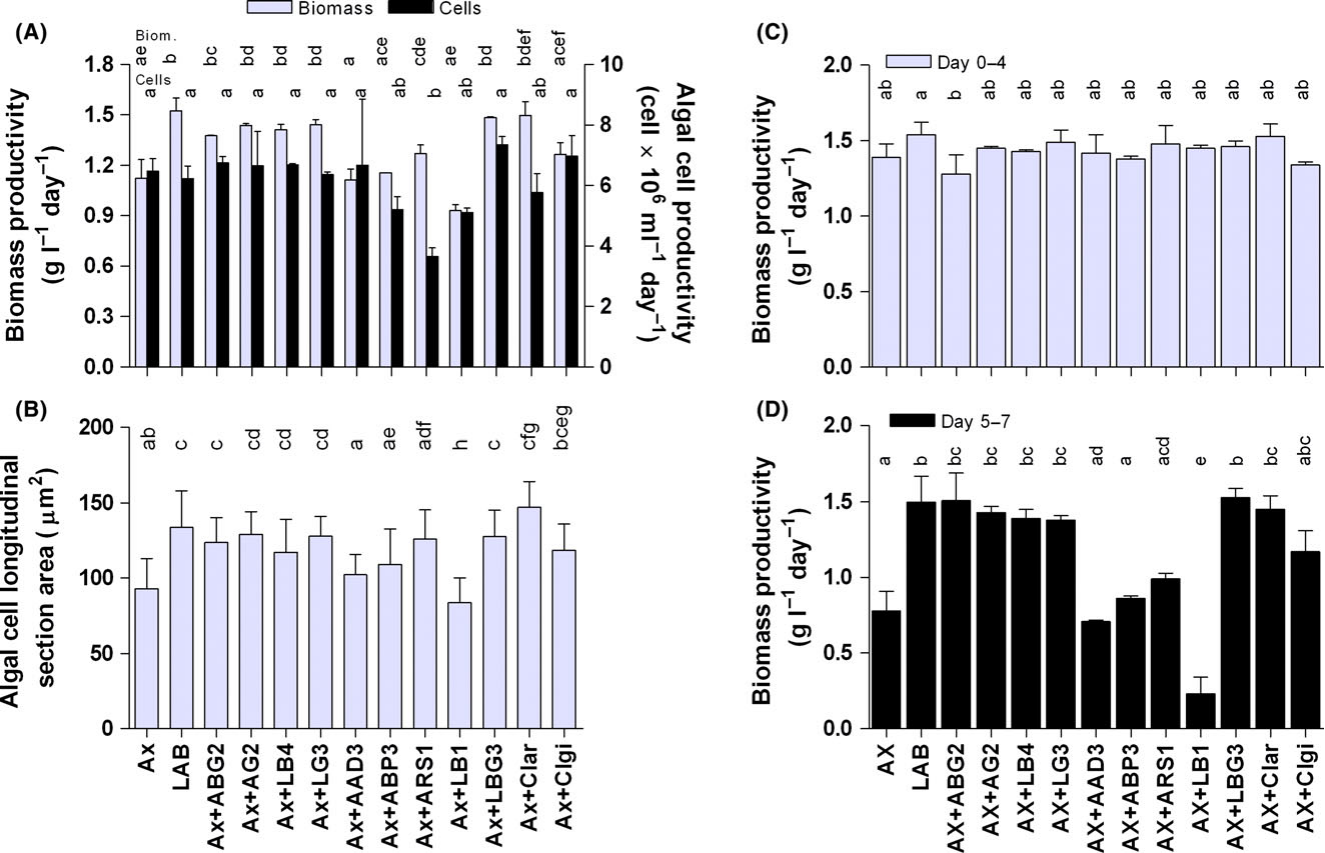
Biomass and algal cell productivities during the seven day cultivation (A), algal cell dimension estimated as surface area of the cell longitudinal section at the end of the trial (day 7) (B), biomass productivity split between the period of nutrient sufficiency (days 0–4) (C) and nutrient depletion (days 5–7) (D), of *Tetraselmis suecica* F&M‐M33 cultures (AX, axenic; LAB, laboratory; AX+ bacterial ID#, axenic culture co‐cultivated with single bacteria) grown in 50 ml bubble tubes. The taxonomic identification of the bacteria indicated here by their codes is reported in Table 3. Error bars indicate standard deviation. The same letters above the bars for each group of data indicate non‐significant difference (*P* > 0.05).

Bacterial isolates were all obtained from *T. suecica* F&M‐M33 phycosphere (LAB and OUT cultures) except two isolates from seawater samples (see Experimental procedures, Table 3). Subcultures of the axenic alga were inoculated with single bacterial isolates to verify the effect of each bacterium on algal growth. The growth of the co‐cultures with single bacteria was compared with that of AX and LAB. The best performance in terms of biomass productivity was obtained by LAB (used as positive control), while among the co‐cultures with single bacteria, the best productivity was achieved with those bacteria always found associated with the alga (*Leewenhoekiella* sp. strain AG2, *Muricauda aquimarina* strain LG3, *Mesorhizobium* sp. strain LB4, *Ponticoccus* sp. strain ABG2) (Fig. 3A). Also, the co‐cultures with the environmental *Nautella italica* strain CIar and with *Roseivivax halotolerns* strain LBG3, associated only with the LAB culture, attained a productivity similar to LAB (Fig. 3A). All these cultures reached productivities of 1.4–1.5 g l^−1^ day^−1^, significantly higher (*P* < 0.05) than the axenic (1.1 g l^−1^ day^−1^), which showed the lowest productivity, except for the co‐culture with the Rhizobiales bacterium LB1 (0.9 g l^−1^ day^−1^). When considering biomass productivity during the first four days, in which nutrients were still available, no significant differences were observed between the axenic and the co‐cultures (Fig. 3C). After nutrient depletion (days 5–7), the productivity of the axenic culture halved compared with the first four days. In this second nutrient‐depleted phase, four co‐cultures (Caulobacteraceae isolate ABP3, *Porphyrobacter* sp. strain AAD3, *Porphyrobacter sanguineus* strain ARS1, and *Alteromonas macleodii* strain CIgi) showed similar productivities compared with AX, six (LAB, *Mesorhizobium* sp. strain LB4, *Ponticoccus* sp. strain ABG2, *Muricauda aquimarina* strain LG3, *Leewenhoekiella* sp. strain AG2, *Roseivivax halotolerns* strain LBG3 and *Nautella italica* strain CIar) showed significantly higher productivities and only the co‐culture with the Rhizobiales bacterium LB1 showed a significantly lower productivity (Fig. 3D).

The differences in productivity among the cultures faded when considering cell number, and similar values were attained by AX and LAB (Fig. 3A). Only the co‐culture with *Porphyrobacter sanguineus* strain ARS1 showed a significantly (*P* < 0.05) lower number of cells produced per unit volume and time compared with AX (Fig. 3A).

These data clearly show that the increase in biomass concentration prompted by the bacterial inoculum is not due to an increase in algal cell number. On the other hand, the increase in bacterial number (Table 1) during the experiments is not sufficient to explain the increase in biomass. In fact, considering that the weight of the inoculated bacteria varied between 63 (*Porphyrobacter sanguineus* strain ARS1) and 260 fg (Rhizobiales bacterium LB1) per cell (see Experimental procedures, Table 3), the contribution of bacteria to the final biomass weight ranges from <0.1 (*Ponticoccus* sp. strain ABG2, *Leewenhoekiella* sp. strain AG2, *Muricauda aquimarina* strain LG3, *Alteromonas macleodii* strain CIgi) to 0.8% (LB4). Even considering that plate counts may have underestimated the total bacterial number, the contribution of bacterial biomass would be in any case negligible. The difference between the productivity of *T. suecica* in terms of dry biomass is mostly due to the difference in the algal cell dimensions. This difference clearly emerges from the comparison between the average surface area of cell longitudinal sections in AX and that of the cells in the cultures inoculated with the bacterial isolates (Fig. 3B). In all the co‐cultures, except those with Rhizobiales isolate LB1, Caulobacteraceae isolate ABP3 and *Porphyrobacter* sp. strain AAD3, the algal cells were significantly bigger than in AX (Fig. 3B). After an initial decrease in dimension following active cell division, cell started to become larger and usually reached their maximum increase in the second nutrient‐depleted phase from day 5 to day 7 (data not shown).

**Table 1:** Bacterial cell counts in *Tetraselmis suecica* F&M‐M33 axenic culture (AX), laboratory culture (LAB) and unibacterial cocultures determined at the start and at the end of the growth period, and percentage contribution to the total (algal + bacterial) biomass weight at the end of the trial. The taxonomic identification of the bacteria indicated here by their codes is reported in Table 3. nc = not calculable

Culture	Bacterial concentration		Estimated contribution to total biomass production
	Start	End	
	CFU × 10^6^ ml^−1^	CFU × 10^6^ ml^−1^	%
AX	0	0	
LAB	11 ± 13	517 ± 112	nc
AX + LB1	0.6 ± 0.2	123 ± 29	0.5
AX + LB4	1.3 ± 0.2	414 ± 83	0.8
AX + LBG3	1.1 ± 0.1	526 ± 60	0.3
AX + LG3	0.1 ± 0.0	14 ± 5	< 0.1
AX + AAD3	35 ± 25	200 ± 61	0.2
AX + ABG2	0.3 ± 0.1	1.7 ± 0.8	< 0.1
AX + ABP3	0.2 ± 0	112 ± 22	0.2
AX + AG2	0.1 ± 0	14 ± 5	< 0.1
AX + ARS1	25 ± 3	124 ± 34	0.1
AX + CIar	1.7 ± 0.7	138 ± 15	0.1
AX + CIgi	0.8 ± 0.1	1.1 ± 0.2	< 0.1

### Influence of vitamins on the growth of axenic and xenic *Tetraselmis suecica* F&M‐M33 cultures

Axenic *T. suecica* F&M‐M33 cultures were grown with and without vitamins in 60 ml shaken bottles for about 3 weeks. At the end of the trial, cultures reached concentrations of 1.6–1.8 g l^−1^. Productivity, both expressed as biomass and cell number, of axenic cultures of *T. suecica* F&M‐M33 in vitamin‐added and vitamin‐deprived media was not significantly different (*P* > 0.05). With vitamins, 79.6 ± 9.0 mg l^−1^ day^−1^ and 0.20 ± 0.04 cells × 10^6^ ml^−1^ day^−1^ were attained, whereas without vitamins productivity reached values of 78.2 ± 6.6 mg l^−1^ day^−1^ and 0.23 ± 0.03 cells × 10^6^ ml^−1^ day^−1^. Analogous results were obtained for the algal cell dimensions: an average longitudinal section surface area of 87.1 ± 8.3 μm^2^ was obtained with vitamins and 99.6 ± 13.5 μm^2^ without vitamins.

Co‐cultures with single bacteria or with the whole community (LAB) were also tested in the same system. Biomass productivity in vitamin‐deprived and vitamin‐added media did not show significant differences, except for the co‐culture with *Leewenhoekiella* sp. strain AG2 that produced less biomass without vitamins (Table 2). No significant differences were obtained for productivity measured as cell number in the two culture media for all the cultures (data not shown). Cell dimensions were never significantly different, although bigger cells (difference was not significant because of the high data variability) were observed in the vitamin‐added medium for *Leewenhoekiella* sp. strain AG2 (data not shown).

**Table 2:** Biomass productivity of axenic (AX), laboratory (LAB) and unibacterial cocultures of *Tetraselmis suecica* F&M‐M33 performed in vitamin‐added and vitamin‐free culture media. The ratio of bacterial concentration at the end compared with the start of the trial is also reported for both culture media. For the same parameter, along the row, the same superscript letter indicates significant difference (*P* < 0.05). No significant differences for biomass productivity were found along the columns, except for AG2 in vitamin‐free medium. The taxonomic identification of the bacteria indicated here by their codes is reported in Table 3. na = not applicable

Culture	Biomass productivity		Bacterial number ratio	
	mg l^−1^ d^−1^		End/start	
	+ Vitamins	− Vitamins	+ Vitamins	− Vitamins
AX	79.6 ± 9.0	78.2 ± 6.6	na	na
LAB	61.8 ± 5.5	53.0 ± 4.2	13.1 ± 1.9	10.1 ± 3.7
AX + ABG2	51.3 ± 12.5	47.0 ± 12.0	5.0 ± 0.2	3.9 ± 1.2
AX + AG2	52.5 ± 16.0^a^	18.0 ± 0.6^a^	1.6 ± 0.5	1.2 ± 1.0
AX + LB4	53.7 ± 20.0	56.7 ± 25.6	3.8 ± 0.4	1.5 ± 0.6
AX + LG3	55.5 ± 8.8	40.0 ± 12.1	3.8 ± 0.6^a^	0.2 ± 0.1^a^
AX + AAD3	83.9 ± 8.3	74.4 ± 15.7	8.8 ± 2.9	3.2 ± 1.0
AX + LB1	77.8 ± 22.0	67.1 ± 24.9	5.9 ± 2.4	6.7 ± 2.8
AX + LBG3	67.5 ± 4.6	56.5 ± 11.9	5.1 ± 1.0^a^	0.9 ± 0.1^a^
AX + CIar	49.5 ± 8.2	52.7 ± 9.8	4.5 ± 1.7	2.8 ± 2.1

It is interesting to note that, on average, at the end of the experiments, a lower number of bacteria was observed in the cultures without vitamins (data not shown) and the ratios between final and initial concentrations were generally lower than those in the vitamin‐added medium, although differences were not significant except for *Muricauda aquimarina* strain LG3 and *Roseivivax halotolerans* strain LBG3 (Table 2).

## Discussion

The presence in cultures of *T. suecica* F&M‐M33 of the whole bacterial community associated with a laboratory culture or with a mass culture kept outdoors year‐round and sampled in autumn (Biondi *et al*., 2017) led to an increase in both cell size and dry weight, but not of cell number, compared with the axenic algal culture. A similar behaviour was observed with the inoculation of single bacterial isolates into axenic cultures when the co‐cultivated bacteria (*Mesorhizobium* sp. strain LB4, *Muricauda aquimarina* strain LG3, *Ponticoccus* sp. strain ABG2 and *Leeuwenhoekiella* sp. strain AG2) were those always found associated with the culture, both in the laboratory and outdoors. To our knowledge, this is the first report on the influence of bacteria on the growth of *T. suecica* cultures. Arora *et al*. (2012) investigated the influence of bacteria on cultures of *Tetraselmis indica* and found that the whole bacterial community showed a positive effect on algal growth (expressed as cell number) compared with the axenic culture. The addition of single bacterial isolates to the axenic culture led to different results: *Acinetobacter* and *Raugeria* enhanced, whereas *Pseudomonas* reduced algal growth (Arora *et al*., 2012). Meseck *et al*. (2007) studied the influence on *T. chuii* of the addition of a bacterial community obtained from a *Tetraselmis striata* culture. The bacteria strongly reduced algal growth compared with the axenic culture. The authors suggested competition for ammonium to be the cause of the reduced growth (Meseck *et al*., 2007). Park *et al*. (2017) evaluated the effect of addition of each of the 26 bacterial isolates obtained from *T. striata* phycosphere to an axenic culture and studied the two isolates (*Pelagibaca bermudensis* and *Stappia* sp.) most effective in promoting *T. striata* growth. *P. bermudensis* was more effective than *Stappia* in algal growth stimulation (Park *et al*., 2017).

The data obtained in our study show that the effect of added bacteria varies according to the algal culture growth phase; namely, the effect during the first nutrient‐replete growth phase is none or very limited compared with the effect observed during the subsequent nutrient‐deficient growth phase. The medium provided enough nitrogen and phosphorus to produce 2.5 g l^−1^ of dry biomass with a content of 10% N and 1% P, typical of biomass grown under optimal conditions (nutrient‐replete phase). Further growth was possible with a decreased N content in the biomass (nutrient‐deficient growth phase). During this phase, cells were unable to divide because of the impossibility to synthesize new proteins and nucleic acids and cells accumulate the energy produced by photosynthesis as the storage products – starch in the case of *T. suecica* F&M‐M33 (Bondioli *et al*., 2012).

**Figure 4:**
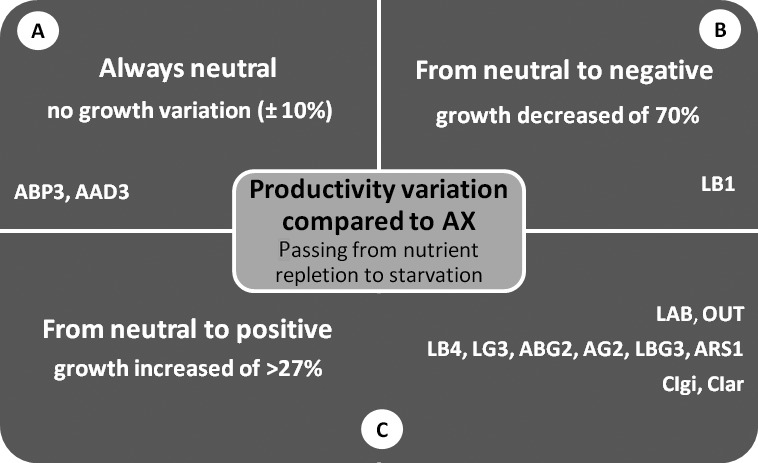
Scheme of the different behaviours of bacterial communities and bacterial isolates with regard to the effect on *Tetraselmis suecica* F&M‐M33 growth in nutrient‐replete and nutrient‐depleted conditions. LAB, bacterial community of the laboratory culture; OUT, bacterial community of the outdoor mass culture; the taxonomic identification of the bacteria indicated here by their codes is reported in Table 3.

A marked response variability among co‐cultures with different bacterial isolates or communities was also found. Three different behaviours were observed (Fig. 4): (i) always neutral, with no growth (biomass productivity) variation compared with the axenic alga in the two growth phases (panel A); (ii) neutral during the first nutrient‐sufficient phase and detrimental during the nutrient‐depleted growth phase (panel B); and (iii) neutral during the first nutrient‐sufficient phase and beneficial during the nutrient‐depleted growth phase (panel C).

Only one strain, LB1 (belonging to the Rhizobiales group), showed a detrimental effect during the nutrient‐depleted phase of growth (B), while none showed a beneficial effect on algal growth during the nutrient‐sufficient phase, although an increase of 17% was attained with the LAB community. The two communities and eight of eleven co‐cultivated bacterial isolates showed no effect on *T. suecica* F&M‐M33 growth when nutrients were present, but increased growth compared with the axenic culture after that the nutrients were exhausted (C). The eight bacteria included those found to be always associated with the alga (Biondi *et al*., 2017). Finally, only two co‐cultivated bacteria (ABP3 and AAD3) were always neutral for algal growth (A). Interestingly, two isolates (AAD3 and ARS1) belonging to *Porphyrobacter* showed a different behaviour, with the first being always neutral and the second increasing *T. suecica* F&M‐M33 growth during the nutrient‐depleted phase. On the contrary, the two isolates belonging to the *Flavobacteriales* (*Leewenhoekiella* sp. strain AG2 and *Muricauda aquimarina* strain LG3) and the three isolates from the *Roseobacter* clade (*Ponticoccus* sp. strain ABG2, *Roseivivax halotolerans* strain LBG3 and *Nautella italica* strain CIar) showed the same behaviour (increased algal growth under nutrient depletion).

In the literature, Flavobacteriales are reported to have different types of interaction with algae cultures, from indifference to growth stimulation, to lethal toxicity (Fukami *et al*., 1997; Suminto and Hirayama, 1997). The same behaviour is reported for strains belonging to the genus *Muricauda*, which enhanced *Dunaliella* biomass production and nitrogen incorporation (Le Chevanton *et al*., 2013) and promoted growth in mixotrophic cultures of *T*. *chuii* and *Cylindrotheca fusiformis* (Han *et al*., 2016). Nevertheless, *Muricauda* is also reported to inhibit *Nannochloropsis gaditana* (Han *et al*., 2016) and kill *Skeletonema* (Shi *et al*., 2013). Strains of the genus *Mesorhizobium* have been shown to provide vitamin B_12_ to cultured algae (Kazamia *et al*., 2012; Grant *et al*., 2014), but this is probably not the reason for increased growth of *T. suecica* F&M‐M33 co‐cultivated with *Mesorhizobium* sp. strain LB4, as vitamins (including B_12_) were added to the culture medium. It is also reported that isolates of the genus *Mesorhizobium* can provide nitrogen through nitrogen fixation (Fuentes *et al*., 2016), which could be an explanation of our results. Isolates of *Alteromonas* showed a different and apparently contradictory behaviour on *Dunaliella* cultures (Le Chevanton *et al*., 2016). When grown in batch, a positive effect emerged, in spite of a final condition of nitrogen depletion, which can be explained by a rapid uptake of the available dissolved nitrogen by the alga and bacterial growth based on the use of dissolved organic nitrogen released by the alga; on the contrary, a continuous regime under nitrogen limitation showed a detrimental effect on growth, implying that bacteria compete with algae for dwindling nitrogen and do not use dissolved organic nitrogen (Le Chevanton *et al*., 2016). Our results with *Alteromonas macleodii* strain CIgi are in accordance with those obtained by these authors with the batch culture. The *Roseobacter*‐clade bacteria are aerobic anoxygenic phototrophs (which also include *Porphyrobacter*) that can use energy from light while growing on organic substances as carbon source (Koblížek, 2015). Members of this clade are reported to favour algal growth (Grossart and Simon, 2007; Seyedsayamdost *et al*., 2011; Luo and Moran, 2014; Park *et al*., 2017), although some representatives are able to turn the mutualistic relationship into a parasitic one, lysing algal cells to feed on the intracellular substances, when molecules signalling algal cell ageing are released (Seyedsayamdost *et al*., 2011). A stimulating effect of the *Roseobacter*‐clade representative *Pelagibaca bermudensis* on *T. striata* growth was reported under P‐limitation, possibly implying a P recycling activity of this bacterium (Park *et al*., 2017). Grossart and Simon (2007) reported growth stimulation of *Thalassiosira rotula* by a *Roseobacter*‐clade isolate in F/2 medium and inhibition in the more oligotrophic F/10, which is not in accordance with the behaviour shown by our isolates on *T. suecica* F&M‐M33; however, our cultures were performed at very high cell density (about 10 g l^−1^ at the end of the trials). No literature on the effect of the genera used in this work (*Roseivivax*,* Ponticoccus*) on algal growth is available, except for a report on a *Nautella* strain epiphytic on a red macroalga (*Delisea pulchra*) becoming pathogenic under certain conditions, such as reduced host defence status following temperature stress (Fernandes *et al*., 2011).

Among the many ways in which bacteria can positively influence algal growth, one of the most commonly reported is the production of vitamins for which the alga is auxotrophic, especially vitamin B_12_ (Croft *et al*., 2005; Amin *et al*., 2012). *T. suecica* F&M‐M33 did not seem to be affected by the lack of vitamins, as the axenic culture grew as biomass (up to concentrations of 1.8 g l^−1^) and cell number in the same way in both vitamin‐supplemented and non‐supplemented media. Croft *et al*. (2005) found that *Tetraselmis verrucosa* is vitamin B_12_ independent. No data are available in the literature on *T. suecica* auxotrophy for one or more vitamins. De Roeck‐Holtzhauer *et al*. (1991) reported rather high contents of thiamine (627 μg g^−1^) and vitamin B_12_ (9 μg g^−1^) in *T. suecica*, but the culture was not axenic. Park *et al*. (2017) found a similar growth of *T. striata* in cultures grown with normal and limiting concentration of vitamin B_12_. In this work, the presence of the whole bacterial community (LAB), as well as the addition of the single bacteria, led to similar *T. suecica* F&M‐M33 growth with or without vitamins except for *Leeuwenhoekiella* sp. strain AG2 (better growth with vitamins). In terms of bacterial growth, the differences observed, showing generally a lower bacterial growth in vitamin‐free medium, were not statistically significant, except in the case of *Muricauda aquimarina* strain LG3 and *Roseivivax halotolerans* strain LBG3. This suggests the following explanations: (i) the bacterial isolate needs one or more of the vitamins usually provided with the culture medium for an optimal growth and (ii) the alga in the absence of vitamins excretes different carbon sources or growth factors that may be detrimental to bacteria. The bacterium with the lowest productivity in vitamin‐free medium was *Muricauda aquimarina* strain LG3 (ratio between cell number at the end and start of the trial was 0.2). Some strains of this genus lack the genes for thiamine synthesis (Monteverde *et al*., 2015); this could be a possible explanation of our results. The other isolate of Flavobacteriales, *Leeuwenhoekiella* sp. strain AG2, performed similarly in vitamin‐added and vitamin‐free medium. *Roseobacter*‐clade representatives are reported to be able to synthesize vitamin B_12_ (Tang *et al*., 2010), but only about half are able to synthesize vitamins B_1_ and B_7_ (Luo and Moran, 2014). An auxotrophy for these latter vitamins may explain the different behaviour of *Roseivivax halotolerans* strain LBG3 (significant decrease in bacterial number during growth in vitamin‐free medium) compared with *Nautella italica* strain CIar and *Ponticoccus* sp. strain ABG2 (similar bacterial number in both media).

To conclude, the interactions between *T. suecica* F&M‐M33 and its associated bacteria are complex and may lead to different growth behaviours of the consortium according to culture conditions. In general, the whole bacterial community has a positive effect on algal growth, although the more stable LAB community showed a higher beneficial effect compared with the community modified by 8 months of permanence outdoors. The beneficial effect on algal growth, seen also for several of the single bacteria tested and especially for those more strictly associated with the alga, was clearly shown during the nutrient‐depleted phase of growth, suggesting a role in nutrient recycling for the associated bacteria. Further investigation of these relationships and a deeper understanding of algal mass culture dynamics, both indoors and outdoors, would be very useful to advance the exploitation of this genus for aquaculture feeds, nutraceutical, and bioenergy applications. In particular, it would be of great interest to establish whether the positive effect of bacteria (with less stable communities than in the laboratory) on growth during nutrient starvation is present and important also in the conditions usually applied in large‐scale algal plants for biofuel production, where nutrient starvation is performed at much lower culture densities, rarely exceeding 1 g l^−1^ in raceway ponds and about 3 g l^−1^ in photobioreactors. Although the positive effects of bacterial communities observed in nutrient‐replete cultures were not statistically significant, bacterial community changes caused by changes in the main cultural parameters could strongly influence the culture performances even under nutrient sufficiency. Last, but not least, the bacterial population may also influence the amount and quality of the desired algal product, a field at present almost unexplored.

## Experimental procedures

### Algal culture conditions

#### Laboratory, axenic and outdoor cultures

The laboratory culture of *T. suecica* F&M‐M33 (LAB) was maintained in F medium (Guillard and Ryther, 1962) in 1 l tubes bubbled with a sterile air/CO_2_ mixture (98:2, v:v) under continuous illumination (200 μmol photons m^−2^ s^−1^). All the operations were carried out in sterile conditions.

LAB was used to set up an outdoor culture that was kept under a semicontinuous harvesting regime from February to October. The outdoor culture (OUT) was carried out in a 300 l Green Wall Panel (GWP^®^‐I) photobioreactor (Chini Zittelli *et al*., 2013) at the Istituto per lo Studio degli Ecosistemi of the CNR in Sesto Fiorentino, near Florence (Italy), and operated as described in Biondi *et al*. (2017). In autumn, an aliquot of the culture was collected and taken to the laboratory for experiments. Once in the laboratory, all the operations were performed in sterile conditions.

Axenic *T. suecica* F&M‐M33 cultures (AX) were obtained from Dr. G. Chini Zittelli of the Institute of Freshwater Ecosystems of the CNR (Florence). Cultures were checked by microscopic inspection and then tested for axenicity by plating on Marine Agar (Difco Laboratories, Detroit, MI, USA) and by incubating in F medium supplemented with glucose (2 g l^−1^) and yeast extract (2 g l^−1^) and in freshwater supplemented with the same amounts of glucose and yeast extract. The inocula of all the axenic cultures used throughout the experiments described in the next paragraphs and for the initial preparation of co‐cultures with single bacteria were prepared in 500 ml Erlenmeyer flasks containing 200 ml of culture. These inocula were incubated in an orbital shaker flushed with an air/CO_2_ mixture (95:5, v:v) at a temperature of 25°C, under continuous illumination (250 μmol photons m^−2^ s^−1^) provided by daylight fluorescent tubes.

#### Algae–bacteria interaction experiments

The OUT, LAB and AX cultures of *T. suecica* F&M‐M33 were used to set up trials in 500 ml tubes containing 350 ml of culture, which was bubbled with a sterile air/CO_2_ mixture (98:2, v:v) under a continuous illumination of about 500 μmol photons m^−2^ s^−1^ provided by daylight fluorescent tubes. The growth test was carried out in duplicate in batch regime using F medium with increased nitrogen (250 mg l^−1^), phosphorus (25.5 mg l^−1^) and iron (6.1 mg l^−1^) concentrations, so as to allow a theoretical growth of 2.5 g (dry biomass) l^−1^ considering N as 10%, P as 1% and Fe as 0.2% of the biomass. The cultures were run for 14 days.

To evaluate the effect of single bacteria on algal growth, the axenic algal culture was subdivided into aliquots and each aliquot was added with one bacterial isolate from *T. suecica* F&M‐M33 or from a natural seawater sample. The newly inoculated co‐cultures were incubated in an orbital shaker for a period long enough (1–2 months) to allow the bacteria to associate with the alga. The presence of bacteria was then checked, and the number of CFU was evaluated by plating at different times the cultures on Marine Agar (Biotec, Grosseto, Italy). The cultures obtained were cultivated in 50 ml bubbled tubes (40 ml culture volume). The trial was performed in batch under a continuous illumination of 200 μmol photons m^−2^ s^−1^ provided by metal halide lamps. Axenic and LAB cultures were also tested. All the cultures were performed at least in duplicate. The cultures were carried out in F medium modified as described for the trials in 500 ml tubes. The experiments lasted 7 days.

For experiments on the vitamin role, to eliminate traces of vitamins from the cultures, all the stock cultures to be tested (AX, LAB, axenic added with the single bacteria) were streaked on agarized F medium deprived of vitamins. When colonies developed, a colony was picked up and transferred to liquid medium deprived of vitamins. Once grown, the culture was transferred again to fresh vitamin‐free medium and this latter culture was used to prepare the inoculum for the experimental cultures. The growth test was carried out in batch using F medium. Each culture was set up in both vitamin‐added (biotin B_7_, 0.2 mg l^−1^; cyanocobalamin B_12_, 1 μg l^−1^; thiamine B_1_, 1 μg l^−1^) and vitamin‐free culture medium. The 60 ml bottles were filled up to 10 ml and closed by SILICOSEN^®^ sterile plugs (Hirschmann Laborgeräte, Eberstadt, Germany) to avoid contamination. Light/dark cycles (12 h:12 h) were applied, providing a light intensity of 100 μmol photons m^−2^ s^−1^ by daylight fluorescent tubes. CO_2_ enrichment of the atmosphere in the orbital shaker incubator allowed to maintain pH at about 8 and provided carbon for growth. The experiment was repeated three times and lasted 21 days.

### Analytical determinations

#### Culture concentration

Biomass (microalgae + bacteria) concentration was followed by measuring dry weight according to Guccione *et al*. (2014). Algal concentration was determined by counting cells using a Thoma haemocytometer. Growth during the experiment in 500 ml tubes was followed daily, while in the 50 ml tubes it was determined every 2 days and in the vitamin experiments only at the start and at the end of the culture period. Nitrate nitrogen in bubble tube experiments was determined at the end of the trials by using a spectrophotometric kit (Hanna Instruments, Woonsocket, RI, USA). Algal cell dimensions were determined on micrographs (Nikon Eclipse E200, Nikon, Tokyo, Japan) taken at 400 magnifications, using an image analysis software (Nikon), and these were used to calculate the surface area of the longitudinal section, approximating the alga shape to an ellipse.

#### Biomass composition

Biochemical analysis of the biomass was performed on culture samples collected at the end of the 500 ml tube experiment. The culture samples were centrifuged and lyophilized. The lyophilized biomass was analysed for carbohydrates (Dubois *et al*., 1956), proteins (Lowry *et al*., 1951) and lipids (Marsh and Weinstein, 1966). Ashes were determined by incineration in a muffle furnace at 550°C.

#### Bacterial concentration

Bacterial concentration was determined at the start and at the end of the experiments for all the cultures including AX. To be sure of axenicity of AX cultures, at the end of the experiments, an aliquot of each culture was tested as reported in the ‘Laboratory, axenic and outdoor cultures’ paragraph.

Direct bacterial cell counts and plate counts were performed for 500 ml tubes experiments. For both counts, serial dilutions of the microalgal culture samples were prepared using a sterile isotonic solution. Samples were maintained in agitation for 3 h to allow the flocks to disperse. For total direct cell count, the acridine orange coloration was used. A 5 ml aliquot of an appropriate dilution of the microalgal culture was filtered through 25 mm black polycarbonate filters (Cyclopore, Whatman, Little Chalfont, UK) 0.2 μm in pore size, and stained with an acridine orange solution (10 mg of acridine orange in a solution of 2 ml of 37% formaldehyde and 98 ml of 6.6 mM phosphate buffer, pH 6.8) for 2 min. The filters were washed with 10 ml of sterile deionized water and mounted on microscope slides by adding 20 μl of 0.6% agarose solution. Each slide was examined by epifluorescence microscopy (Nikon Eclipse 400) at magnification 1000, BP 450–490 nm exciter filter, FT 510 nm beam splitter, LP 520 nm barrier filter and counting at least 30 microscope fields per filter.

For plate counts, aliquots of 100 μl of each culture dilution were spread in triplicate on Marine Agar plates, which were incubated at 27°C. The number of colony‐forming units (CFU) was evaluated after 6 days. For experiments of algal growth in 50 ml tubes and for vitamin experiments, the viable cell counts were performed by serially diluting the culture in 96 well microtiters and plating three 20 μl aliquots of each dilution in spots. Colony count was performed under the microscope. Plating the cultures also allowed verification of the presence of only one bacterium in the culture at the end of the experiments and that the bacterium present was the same inoculated in the culture. In case of doubts, the cultures were discarded and repeated.

Bacterial cell weight of the isolated strains used to inoculate the axenic algal cultures was estimated using bacterial cultures in active growth. An aliquot of the bacterial culture was filtered through 0.2 μm pore size filters to determine dry weight; another aliquot was used for viable counts with the spot method and another for total counts by Neubauer haemocytometer (depth of 0.01 mm). The cell weight was estimated by dividing the dry weight value by the number of bacterial cells determined in the total count. The contribution of bacteria to the total biomass (algae + bacteria) weight at the end of the experiment was calculated on the basis of the viable counts performed at the end of the experiment and corrected by the ratio between the total and the viable counts of the bacterial cultures.

### Bacterial strain selection and isolation

The bacteria used in the trials were isolated from the phycosphere of *T. suecica* F&M‐M33 LAB and OUT cultures by plating aliquots of the algae on Marine Agar plates and selecting a certain number of colonies of different morphology according to their abundance. The colonies were streaked until pure cultures were obtained. Different operational taxonomic units were identified by amplified ribosomal DNA restriction analysis (ARDRA) and identified by 16S rRNA gene sequencing (Biondi *et al*., 2017) (Table 3). Besides, two bacteria were isolated from natural seawater collected at Coral Island (Thailand) and identified following the same methods as for the other isolates (Table 3). All the bacteria were maintained in Marine Agar. Besides the two environmental isolates (CIar and CIgi), the other bacteria were selected for the experiment on the basis of the terminal fragment length polymorphism (T‐RFLP) analysis which included results for the whole communities of LAB culture and OUT culture in different seasons as well as for the single bacterial isolates obtained from these cultures (Biondi *et al*., 2017). In particular, the selection included the four isolates (LB4, LG3, ABG2 and AG2), the signal of which was present in the T‐RFLP electropherograms of all the algal cultures sampled (laboratory and outdoors in different seasons), as well as some of the isolates (LB1, LBG3, AAD3, ABP3, ARS1), the signal of which was found in the T‐RFLP electropherogram of only one algal culture and thus might be considered as occasional contaminants (Biondi *et al*., 2017).

**Table 3:** List of the bacterial isolates tested for their effect on *Tetraselmis suecica* F&M‐M33 growth, the origin of the isolate (*T. suecica* F&M‐M33 culture or natural seawater), closest relative with 16S rRNA gene sequencing, GenBank accession number, cell weight and type of relationship with the algal cells

Strain	Origin	Phylogenetic group	Closest relative	Similarity (%)	GenBank accession number	Cell weight (fg)	Association with algal cells
LB1	LAB	Rhizobiales	Rhizobiales bacterium CSQ‐10	100	MF157558	260	Epiphytic
LB4	LAB	Rhizobiales	*Mesorhizobium* sp. VBW011	99	MF157559	200	Epiphytic
LBG3	LAB	Roseobacter clade	*Roseivivax halotolerans* NBRC16686	98	MF157560	64	Epiphytic
LG3	LAB	Flavobacteriales	*Muricauda aquimarina* strain 97A	100	MF157561	86	Epiphytic
AAD3	OUT	Sphingomonadales	*Porphyrobacter* sp. MBIC3897	100	MF157553	69	Free‐living
ABG2	OUT	Roseobacter clade	*Ponticoccus* sp. MBTDCMFRIMab06	99	MF157554	117	Epiphytic
ABP3	OUT	Caulobacterales	Caulobacteraceae bacterium MOLA 378	99	MF157555	156	Free‐living
AG2	OUT	Flavobacteriales	*Leeuwenhoekiella* sp. M56‐8	100	MF157556	176	Epiphytic
ARS1	OUT	Sphingomonadales	*Porphyrobacter sanguineus* NBRC 15763	100	MF157557	63	Free‐living
CIgi	Natural seawater	Alteromonadales	*Alteromonas macleodii* HOT1A3	100	MF157562	79	Free‐living
CIar	Natural seawater	Roseobacter clade	*Nautella italica* LMG 24365	99	MF157563	96	Free‐living

#### Statistical analyses

Experimental results were evaluated by linear regression analysis, one‐way ANOVA followed by Tukey's multicomparative test, or by Student's *t*‐test.

## Conflict of interest

The authors declare no conflicts of interest.
